# Identification of Combinations of Protein Kinase C Activators and Histone Deacetylase Inhibitors that Potently Reactivate Latent HIV

**DOI:** 10.3390/v12060609

**Published:** 2020-06-03

**Authors:** Francesca Curreli, Shahad Ahmed, Sofia M. Benedict Victor, Asim K. Debnath

**Affiliations:** Laboratory of Molecular Modeling and Drug Design, Lindsey F. Kimball Research Institute, New York Blood Center, 310 E 67th Street, New York, NY 10065, USA; SAhmed@nybc.org (S.A.); SVictor@nybc.org (S.M.B.V.); ADebnath@nybc.org (A.K.D.)

**Keywords:** HIV-1, latency, latency reversing agents (LRA), combinations, protein kinase C activators, histone deacetylase inhibitors

## Abstract

Combination antiretroviral therapy (cART) is successful in maintaining undetectable levels of HIV in the blood; however, the persistence of latent HIV reservoirs has become the major barrier for a HIV cure. Substantial efforts are underway in finding the best latency-reversing agents (LRAs) to purge the latent viruses from the reservoirs. We hypothesize that identifying the right combination of LRAs will be the key to accomplishing that goal. In this study, we evaluated the effect of combinations of three protein kinase C activators (prostratin, (-)-indolactam V, and TPPB) with four histone deacetylase inhibitors (AR-42, PCI-24781, givinostat, and belinostat) on reversing HIV latency in different cell lines including in a primary CD4+ T-cell model. Combinations including indolactam and TPPB with AR-42 and PCI produced a strong synergistic effect in reactivating latent virus as indicated by higher p24 production and envelope gp120 expression. Furthermore, treatment with TPPB and indolactam greatly downregulated the cellular receptor CD4. Indolactam/AR-42 combination emerged from this study as the best combination that showed a strong synergistic effect in reactivating latent virus. Although AR-42 alone did not downregulate CD4 expression, indolactam/AR-42 showed the most efficient downregulation. Our results suggest that indolactam/AR-42 is the most effective combination, showing a strong synergistic effect in reversing HIV latency combined with the most efficient CD4 downregulation.

## 1. Introduction

At present, HIV-1 is still an incurable infection. Although combination antiretroviral therapy (cART) represses HIV to undetectable levels, the persistence of latent HIV reservoirs has become the primary barrier to curing HIV [1], and interrupting cART can cause the virus to rebound to pretreatment levels rapidly. Therefore, to keep HIV replication suppressed, infected individuals must commit to lifelong cART. The lifelong treatment with cART is not an acceptable solution to treat HIV/AIDS at either an individual or global scale because of the associated problems such as accumulation of side effects, high cost, and the possibility of non-adherence [2]. As a result, the elimination of replication-competent HIV from the human body (sterilizing cure) or long-term control of HIV-1 in the absence of cART (functional cure) are needed [3].

Latently infected cells harbor integrated proviruses, which are transcriptionally silenced but replication-competent, lack the expression of viral proteins, making them invisible to the immune system. However, following stimulation with agents capable of reversing latency, these cells can express viral proteins [4,5]. It has been reported that the quiescent, central memory CD4+ T-cells are the major source of the HIV reservoir. However, other types of lymphoid cells such as naive CD4+ T-cells, stem memory T-cells, and transitional memory CD4+ T-cells can harbor integrated latent HIV proviruses [4,6]. Over 90% of memory and naive CD4+ T-cells isolated from both lymph node tissue and peripheral blood contain only one integrated HIV-1 DNA molecule [7,8]. The molecular mechanisms involved in the establishment of HIV latency have not yet been fully elucidated because of their complexity and the numerous factors involved. A characteristic of quiescent CD4+ T-cells is the low availability of transcription factors, including NF-Κb and NFAT, due to cytoplasmic sequestration [9]. Moreover, in resting cells, the transcription factors are replaced by transcriptional repressors, which induce epigenetic modifications in the form of de-acetylation and methylation of histones and DNA, increasing the compaction of chromatin and contributing to repression of HIV gene expression, thus, inducing gene silencing [9].

Several therapeutic strategies are being considered to control or eliminate the HIV latent reservoir. One of these strategies known as “shock and kill” consists of two phases: the first phase induces the reversal of HIV latency to reveal the latent reservoir and induce viral production (“shock”), followed by clearance of the cells (“kill”) by cytopathic death induced by the viruses or by a combination of the native or engineered immune response [10,11]. This method employs drugs or small molecules, also called latency-reversing agents (LRAs), to force the reactivation of latent HIV in memory CD4+ T-cells. LRAs are classified based on their targets [12]. Among these, the histone deacetylase inhibitors (HDACis) induce an overall chromatin de-compaction permitting accessibility to the transcription factors and reactivation of latent HIV [13,14]. Protein kinase C activators (PKCas) induce transcription factors such as NF-κB, which binds to HIV-LTR and activates HIV mRNA transcription [12,15,16]. In most reports, the activity of PKCas and HDACis as LRAs has been evaluated mostly as single compounds [14,15,17,18,19]. However, in a few cases, combinations of LRAs were reported [20,21,22]. As mentioned above, the establishment of latency is a complicated process, and numerous factors and cellular mechanisms are involved. Thus, a combination of agents that trigger multiple pathways at the same time should be a more successful way to reactivate the latent virus. In this report, we evaluated the effect of combinations of three PKCas, prostratin, (-)-indolactam V, and TPPB, with four HDACis, AR-42, PCI-24781 (abexinostat), belinostat and givinostat on HIV reactivation. Prostratin, a widely studied PKCa agent and a non-oncogenic phorbol ester, was shown to have tumor-suppressing activity and a variety of biological activities, including antagonizing HIV latency by activating NF-κB and inhibiting de novo HIV infection, most likely because it downregulates cellular receptor CD4 [23,24,25,26]. TPPB, a cell-permeable PKCa [27] and (-)-indolactam V (which has been recently reported to reactivate HIV-1 in ACH-2, U1, and J-Lat cells) [18,19] are two benzolactam derivatives. AR-42 is a novel anticancer drug candidate that potently induces histone acetylation and has been reported to reactivate HIV-1 [17]. Belinostat and givinostat were also previously shown to reactivate latent HIV-1 in U1 and ACH-2 cells [28,29,30]. PCI-24781 is a pan-HDACi, experimental drug candidate for cancer treatment [31]. Here, we reported that the combination of AR-42 and PCI with (-)-indolactam V and TPPB greatly increased HIV reactivation as measured by p24 release in three different cell lines latently infected with HIV-1 (OM-10.1, U1, and ACH-2) and using flow cytometry for Green Fluorescent Protein (GFP) expression in two J-Lat cell lines. These findings were further confirmed using Real-Time quantitative Reverse Transcription- PCR (Real-Time qRT-PCR) which showed that the combination of AR-42 and PCI with (-)-indolactam V and TPPB induced higher amounts of viral RNA with respect to the control, which was treated with phorbol 12-myristate 13-acetate (PMA) (up to 60-fold increase in OM-10.1 and up to 12-fold increase in ACH-2 cells). Moreover, here we showed that the combinations of indolactam and TPPB with AR-42, PCI and givinostat induced high expression of envelope glycoprotein gp120 and downregulation of the cellular receptor CD4 more efficiently than combinations of the same HDACis with prostratin. Finally, we confirmed the activity of one of our most effective combinations, indolactam/AR-42, in a HIV latency model in primary CD4+ T cell.

## 2. Materials and Methods

### 2.1. Cell Lines

The following cell lines were obtained through the NIH AIDS Reagent Program (ARP): Jurkat (E6-1) cells [32], J-Lat 10.6 cells (a GFP-expressing Jurkat cell line infected with the full-length HIV-1 genome with a non-functional Env due to a frameshift) [33], J-Lat A2 cells (a GFP-expressing Jurkat cell line infected with retroviral construct LTR-Tat-IRES-GFP) [33], U1 cells (a subclone of U937, a pro-monocyte cell line chronically infected with HIV-1) [34], ACH2 cells (HIV-1 latent T cell clone with one integrated proviral copy of HIV-1 LAV per cell) [35] and OM-10.1 cells (a promyelocyte cell line with one integrated proviral copy of replication-competent HIV-1 per cell) [36]. The h-PBMCs (human peripheral blood mononuclear cells) were isolated from buffy coats of healthy HIV-1 negative donors obtained from the New York Blood Center (New York, NY, USA) and cultured in RPMI 1640 medium supplemented with fetal bovine serum (FBS) penicillin and streptomycin and induced with 20 U/mL of IL-2 and 5 µg/mL of PHA. The total CD4+ T-cells were isolated from uninduced h-PBMCs with the RosetteSep™ Human CD4+ T Cell Enrichment Cocktail (STEMCELL Technologies, Cambridge, MA, USA) by following the manufacturer’s instructions.

### 2.2. Reagents

PMA (phorbol 12-myristate 13-acetate) was purchased from Biovision (Milpitas, CA, USA). TNFα (Life Technologies, Carlsbad, CA, USA) and Brefeldin A Solution, 1000x, was purchased from Fisher healthcare. Prostratin, givinostat hydrochloride hydrate, and vorinostat or suberoylanilide hydroxamic acid (SAHA) were obtained from Sigma (St. Louis, MO, USA). (-)-indolactam V, TPPB, PCI-24781, and belinostat were purchased from MCE (MedChem Express). AR-42 was purchased from Selleckchem.

### 2.3. Evaluation of HIV-Latency Reversal in Cell Lines

For the initial screening, GFP-expressing J-Lat 10.6 and J-Lat A2 cells were cultured in 96-well round-bottom plates at 10^6^ cells/mL in the presence of serial dilutions of LRAs and incubated for 48 h. Untreated cells and cells treated with PMA were used as controls. GFP expression indicating viral reactivation was evaluated using flow cytometry. In addition, OM-10.1, U1, and ACH2 cells at 10^6^ cells/mL were treated with serial dilutions of the LRAs for 48 h. Untreated cells and cells induced with PMA were used as controls. The supernatants were collected and mixed with an equal volume of 5% Triton X-100 and tested for Gag p24 antigen using sandwich enzyme-linked immunosorbent assay (ELISA). EC_50_ (the concentration of a drug that gives a half-maximal response) was calculated with GraphPad Prism software to determine the ideal dosage to be used for drug combinations.

For the LRA double combination studies, cells were treated with LRAs alone or in combination for 48 h and evaluated for p24 antigen detection as described above to calculate the HIV-1 expression with respect to uninduced cells and cells induced with PMA.

### 2.4. Evaluation of Cytotoxicity

The cytotoxicity of the LRAs in all the cell lines was measured with CellTiter-Blue^®^ cell viability assay (Promega) following the manufacturer’s instructions. Briefly, the cells were plated in a 96-well black tissue culture plate at 10^6^/mL and cultured in the presence of escalating concentrations of LRAs for 48 h. The percent of cytotoxicity and the CC_50_ (the concentration for 50% cytotoxicity) values were calculated with GraphPad Prism software. The cytotoxicity of the LRAs alone or in combination was also evaluated in uninfected Jurkat cells and induced human PBMCs following 48 h treatment with the CellTiter-Blue^®^ cell viability assay.

### 2.5. Real-Time qRT-PCR (Real-Time Quantitative Reverse Transcription PCR) and HIV-1 RNA Analysis

For the cell-associated HIV-RNA study, OM-10.1, U1, and ACH-2 cells at 10^6^/mL were treated with LRAs alone or in combination for 48 h as reported above. Cellular RNA was extracted from cell pellets using ReliaPrep™ RNA miniprep systems (Promega), following the manufacturer’s instructions. Real-time qRT-PCR was carried out in duplicates using TaqMan^®^ Fast Virus 1-Step master mix (Life Technologies, Carlsbad, CA, USA). The set of probe and primers for HIV-1 RNA quantification which amplifies a 199-bp fragment in a conserved region of the subtype B HIV-1 Pol gene (HIV-1 TaqMan probe: 5′-/56-FAM -TTTATCTACTTGTTCATTTCCTCCAATTCCTT/36-TAMSp/-3′, HIV-1-S: 5′-TGGCATGGGTACCAGCACA-3′ and HIV-1-AS: 5′-CTGGCTACTATTTCTTTTGCTA-3′) [37] and the set of primers and probe for GAPDH (GAPDH TaqMan probe: 5′-/56-FAM/AAGGTCATCCCTGAGCTGAAC/36-TAMSp/-3′, GAPDH-S: 5′-GAACATCATCCCTGCCTCTACT-3′ and GAPDH-AS: 5′-ATTTGGCAGGTTTTTCTAGACG-3′) were synthesized by Integrated DNA Technologies, Inc. The results were analyzed using the comparative CT method and expressed as 2^−ΔΔ*C*t^ which is the amount of HIV-RNA normalized to endogenous GAPDH and relative to the control induced with PMA. The graphs show the average of two biological samples plus/minus standard deviation. For the virus-associated HIV-RNA study, the viral RNA was isolated from the culture supernatant using a QIAamp^®^ Viral RNA mini kit (QIAGEN) and following the manufacturer’s instructions. Real-time qRT-PCR was carried out using the PCR primers and Taqman probe for HIV as above. The number of HIV RNA copies/mL was quantified using the HIV RNA quantification standard obtained through the NIH ARP (catalog #3443). The number of HIV RNA copies indicating HIV activation was expressed as the percentage of activation in respect to the control induced with PMA set to 100. The graphs show the average of two biological samples plus/minus standard deviation.

### 2.6. Evaluation of HIV Reactivation by Western Blot

OM-10.1, ACH-2, and U1 cells at 10^6^/mL were treated with LRAs alone or in combination for 48 h. Untreated cells and cells induced with PMA were used as controls. As an additional control, we used OM-10.1 cells treated with 50 ng/mL TNFα. Cellular pellets were lysed with RIPA extraction buffer containing 1× Halt™ Protease Inhibitor Cocktail (Thermo Scientific, Waltham, MA, USA) to extract total cellular proteins. Proteins were quantified using a Pierce™ Coomassie (Bradford) Protein Assay Kit (Thermo Scientific, Waltham, MA, USA). The same volumes of culture supernatants were filtered and ultra-centrifuged for 1 h at 40,000 rpm to concentrate the viral particles. Viral pellets were then processed for protein analysis. The same amounts of viral and cellular proteins were resolved on a NuPAGE Novex 4–12% Bis-Tris Gel (Invitrogen). Proteins were then visualized using immunoblot and immuno-detected with HIV-1 anti-p24 Gag monoclonal antibody (mAb) (NIH ARP). Cellular proteins were also immuno-detected with the housekeeping gene β-actin as a loading control. Proteins were visualized using chemiluminescence, and the bands were quantified using ImageJ software (http://imagej.nih.gov/ij/).

### 2.7. Establishment of HIV Latency in Primary CD4+ T-Cells

The primary cell model of HIV-1 latency was established as previously described [18,38,39] with minor modifications. Briefly, total resting CD4+ T-cells from healthy donors were maintained at 5 × 10^6^/mL in the presence of 100 nM of CCl19. On the third day, the cells were washed and infected with HIV-1_NL4-3_ (100 ng/mL of p24) via spinoculation at room temperature for 2 h at 1200× *g*. After infection, the cells were resuspended at 2 × 10^6^/mL in complete medium supplemented with 20 U/mL of IL-2 and cultured for 7 days. Seven days post-infection cells were either treated with combinations of compounds or left untreated. Additionally, control cells were induced with Dynabeads Human T-Activator CD3/CD28 (Thermofisher). Following 48 h treatment, the cells were washed with PBS and incubated with 4 µg/mL of anti-HIV-1 gp120 mAb NIH45-46 G54W (NIH ARP) for 20 min. Cells were then washed 2× with PBS and incubated with goat anti-human IgG (H + L) cross-adsorbed secondary antibody, Alexa Fluor 488, anti-CD4 mAb (S3.5), and anti-CD3 mAb (UCHT1) (Invitrogen) for 30 min. Cells were washed 3× with PBS, and the gp120 expression was evaluated using flow cytometry.

### 2.8. Detection of gp120 Expression

OM-10.1 and ACH-2 cells were plated at 10^6^/mL in 96-well plates and treated with LRAs alone or in combination. Untreated cells and cells induced with PMA or TNFα were used as controls. Following 24 h and 48 h incubation, the cells were analyzed for gp120 expression. Briefly, the plates were spun to pellet the cells and remove the supernatant. Cells were washed with PBS twice. OM-10.1 cells and ACH-2 cells were incubated with 1 µg/mL and 2 µg/mL, respectively, of anti-HIV-1 gp120 mAb VRC03 (NIH ARP) for 30 min. Cells were then washed with PBS and incubated with goat anti-human IgG (H + L) cross-adsorbed secondary antibody, Alexa Fluor 488, for 20 min. Cells were washed 3x with PBS, and the gp120 expression was evaluated using flow cytometry by gating on live cells.

### 2.9. Drug Interaction Analysis

The synergism/antagonism effect of drug combinations was analyzed with the Bliss independence model [40] using the formula for probabilistic independence EA + EB − EAEB, where EA = effect of drug A, EB = effect of drug B, and 0 ≤ EA ≤ 1 and 0 ≤ EB ≤ 1. The resulting combination index (CI) can be calculated as:

CI = (EA + EB-EAEB)/EAB,

CI < 1 indicates synergism,

CI = 1 indicates additivity,

CI >1 indicates antagonism.

## 3. Results

### 3.1. Evaluation of the Efficacy and Cytotoxicity of the LRAs

**Figure 1:**
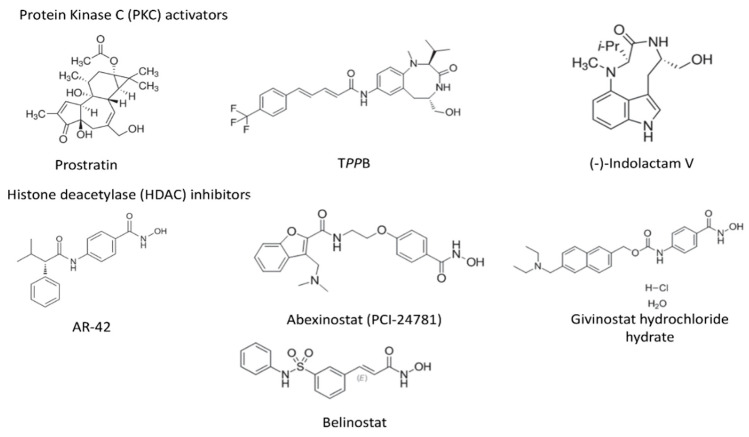
Chemical structure of latency-reversing agents (LRAs).

As first step, we performed dose-response experiments to determine the EC_50_ (50% effective concentration) and CC_50_ (50% cytotoxic concentration) of the seven compounds (Figure 1) including three PKCas, namely, prostratin (Pro), (-)-indolactam V (indo) and TPPB, capable of activating the NF-κB signaling pathway and four HDACis, AR-42, PCI-24781, givinostat (givino) and belinostat (belino) which induce de-compaction of chromatin and higher accessibility for the transcription factors. It has been reported that patients carry viral DNA integrated into different chromosomal locations [41,42]; therefore, a good LRA should be capable of reactivating viral expression in different cell models. To perform our study, we selected three cell lines latently infected with competent HIV-1 (OM-10.1, U1, and ACH-2) and two Jurkat-derived J-Lat cell lines that express GFP upon activation, J-Lat Tat-GFP (A2) and J-Lat full length (10.6) cells. We evaluated the dose-response by quantification of the expression of HIV-1 Gag protein p24 in the supernatant of OM-10.1, U1, and ACH-2 cells and by quantification of GFP expression using flow cytometry in the J-Lat cells. The EC_50_ values obtained for the compounds targeting PKC tested in OM-10.1, U1, and ACH-2 cells were in the range of 0.07–0.41 µM, while the values obtained with both J-Lat cell lines were slightly higher (0.2–0.87 µM) (Table 1). Indolactam displayed the lowest EC_50_ (0.09–0.32 µM) in four of the cell lines tested (OM-10.1, U1, J-Lat 10.6, and J-Lat A2), while TPPB was the most effective in ACH-2 cells with a calculated EC_50_ of 0.07 µM. Prostratin had the highest EC_50_ compared to the other PKCas in all the cell lines tested (0.3–0.87 µM). The HDACis had EC_50_ in the range of 0.24–0.94 µM. AR-42 was the most effective compound (EC_50_: 0.24–0.55 µM), while belinostat had the highest EC_50_ (0.44–0.94 µM). The cytotoxicity assay showed that the PKCas did not induce toxicity at the higher concentration tested (CC_50_ > 8 µM). Consistent with previous reports [43,44], the HDACis exhibited high cytotoxicity. All the cell lines tested, except for the U1 cell line (CC_50_ > 4 µM), were highly sensitive to these compounds, as shown by their CC_50_ (0.3–1.4 µM). Based on these results, we selected non-toxic concentrations (0.5 µM for the PKCas and 0.3 µM for the HDACis) for further studies.

### 3.2. Combinations of PKCas and HDACis Do not Affect the Viability of Uninfected Cells

**Figure 2:**
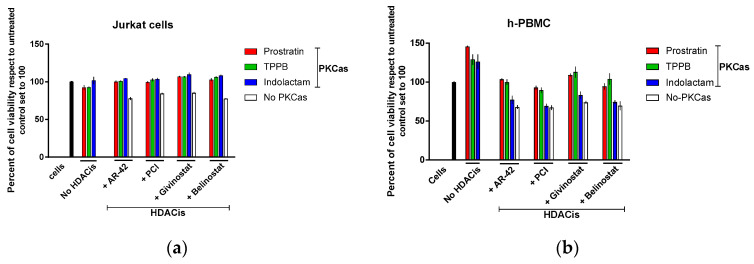
Cellular toxicity assessment of combinations of LRAs in uninfected cells. The effect of combinations of LRAs on cell viability in Jurkat cells (**a**) and human PBMCs (**b**) following 48 h treatment was measured with CellTiter-Blue^®^ Cell Viability assay. Black columns represent untreated cells; red columns are cells treated with prostratin in combination with or without HDACis; green columns are cells treated with TPPB in combination with or without HDACis; blue columns are cells treated with indolactam in combination with or without HDACis; and white columns are cells treated with a single HDACi. Data are shown as mean ± S.D. of three independent experiments.

To exclude toxicity due to viral expression, we also evaluated the direct impact of the combinations of LRAs on cell viability with cells that do not carry integrated HIV proviruses. To this end, we treated Jurkat (E6-1) cells and human PBMCs (h-PBMCs) with the LRAs alone or in combination for 48 h (Figure 2). Untreated cells were used as control. The PKCas alone did not affect the viability of Jurkat cells, which was >93%, while the cells treated with the HDACis alone had viability in the range of 78–85% with respect to the untreated control (Figure 2a). Moreover, the combinations of PKCas and HDACis did not induce toxicity, and the viability was 100%. The PKCas alone did not affect viability in h-PBMCs (Figure 2b) as well, but by contrast, they induced proliferation (26–45% with respect to the untreated control). The HDACis alone had a bigger impact on cell viability. The viable cells were 67–74% compared to untreated control. Finally, the cell viability in the samples treated with combinations of LRAs was higher (69–109%) than the viability detected in the cells treated with HDACis alone, suggesting that the PKCa compounds were attenuating or masking the unfavorable toxic effect of the HDACis. However, we do not know the exact mechanism of the attenuation of the toxic effects. More experiments will be necessary to completely elucidate these findings.

### 3.3. Combinations of LRAs Reactivate HIV Expression in Latently Infected Cells

**Figure 3:**
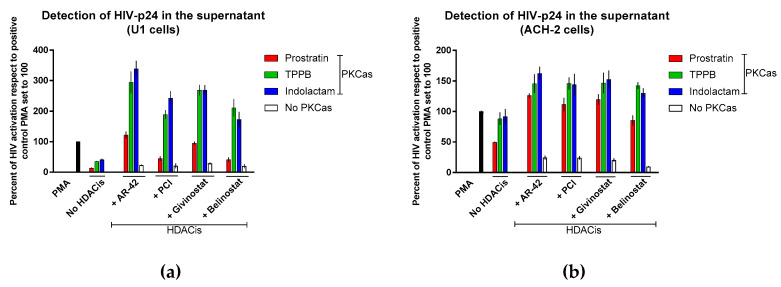
Effect of combinations of LRAs on the reactivation of latent HIV. The HIV reactivation was measured using ELISA: (**a**) U1 cells and (**b**) ACH-2 cells. Cells were treated with LRAs alone, or in combination, for 48 h, cells induced with PMA were used as a control. The HIV expression was measured by detection of the viral Gag p24 released in the supernatant and expressed as a percentage of the positive control PMA set to 100 (black columns). Red columns represent cells treated with prostratin in combination with or without HDACis; green columns are cells treated with TPPB in combination with or without HCDAis; blue columns are cells treated with indolactam in combination with or without HDACis; and white columns are cells treated with a single HDACi. Data are shown as mean ± S.D. of three independent experiments.

We further evaluated the effect of combinations of LRAs by treating cells latently infected with competent HIV-1 (U1 and ACH-2) and quantifying the expression of HIV-1 Gag protein p24 in the supernatant using sandwich ELISA. In U1 cells, we observed that all the LRA combinations induced activation of HIV latency in a synergistic manner (Figure 3a). The best results were achieved by exposing the cells to indolactam/AR-42 and TPPB/AR-42 combinations. These combinations induced about 3-fold higher HIV reactivation than the control that was treated with PMA (HIV reactivation was 339% and 295%, respectively). The combinations of givinostat with indolactam and TPPB were also highly effective, producing a 2.7-fold increase of HIV reactivation with respect to the control cells. The combinations of PCI and belinostat with indolactam and TPPB were somewhat less effective, producing a 1.7–2.4-fold increase of HIV reactivation when compared to the control. By contrast, the combination of prostratin with the four HDACis was less effective. When combined with AR-42 and givinostat, HIV reactivation was similar to the positive control, and showed 41% and 45% activation when combined with PCI and belinostat, respectively. Exposure of ACH-2 cells to the combinations of LRAs induced HIV reactivation synergistically. The best results were achieved with the combination of AR-42 with indolactam; however, the percentage of HIV activation was lower than what we observed in U1 cells (Figure 3a). Of note, apart from prostratin/belinostat, all the combinations induced HIV expression higher than what was observed in the positive control treated with PMA (the HIV activation was 11–26% higher than positive control).

**Figure 4:**
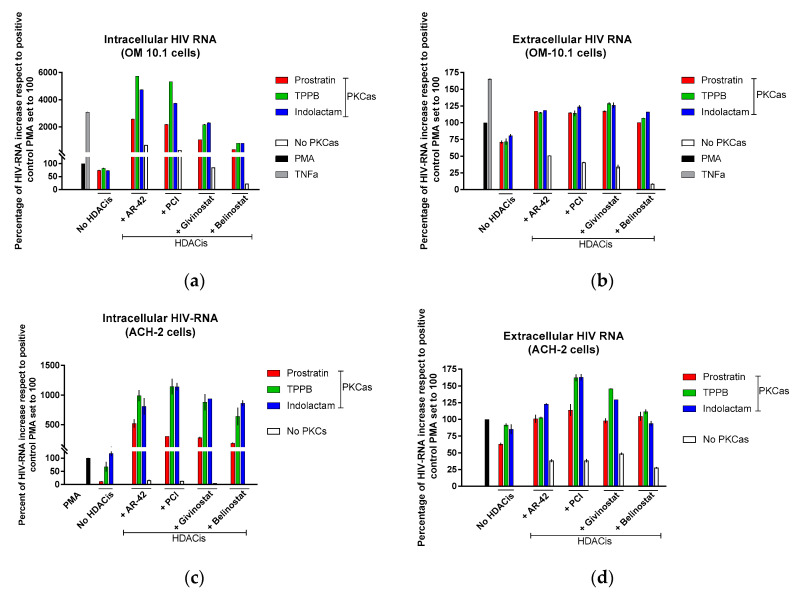

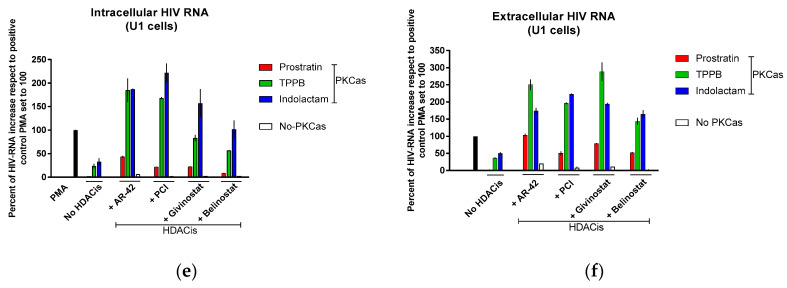
Combinations of LRAs reactivate latent HIV measured using HIV RNA quantification. (**a**,**b**) OM-10.1, (**c**,**d**) ACH-2, and (**e**,**f**) U1 cells were treated with LRAs alone or in combination for 48 h. OM-10.1 positive control cells were treated with PMA or TNFα. U1 and ACH-2 positive control cells were treated with PMA. The cell-associated (intracellular) and virus-associated (extracellular) HIV RNA was detected using qRT-PCR and expressed as a percentage of the positive control PMA set to 100 (black columns). Gray columns represent cells treated with TNFα; red columns are cells treated with prostratin in combination with or without HDACis; green columns are cells treated with TPPB in combination with or without HDACis; blue columns are cells treated with indolactam in combination with or without HDACis; and white columns are cells treated with a single HDACi. Data are shown as mean ± S.D. of three independent experiments.

**Figure 5:**
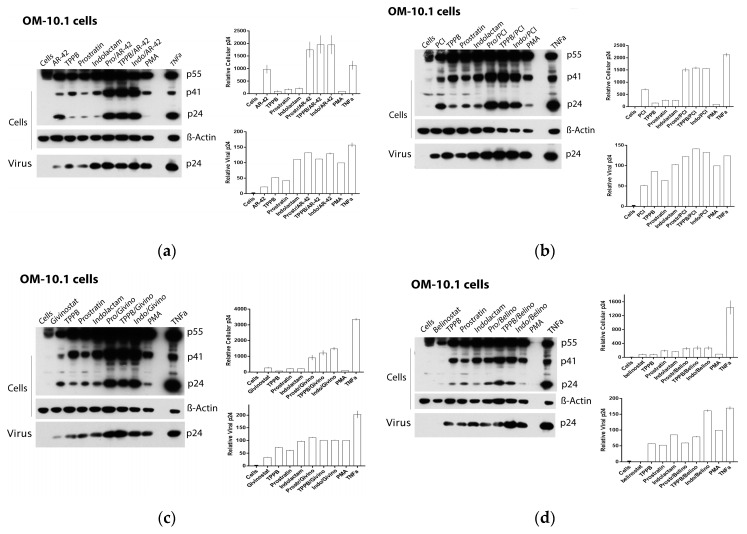
Effect of combinations of LRAs on the reactivation of latent HIV in OM-10.1 cells. Immunoblot analysis of cell lysates and viral lysates and related densitometry analysis. β-actin was used as a loading control. The densitometry analysis of the cell-associated Gag p24 is relative to β-actin and expressed as a percentage of the positive control PMA set to 100. The densitometry analysis of the virion-associated Gag p24 is relative to the untreated control cells and expressed as a percentage of the positive control PMA set to 100. Immunoblots are representative of two independent experiments. A representative study of (**a**) AR-42, (**b**) PCI, (**c**) givinostat, and (**d**) belinostat in combination with the PKCas.

**Figure 6:**
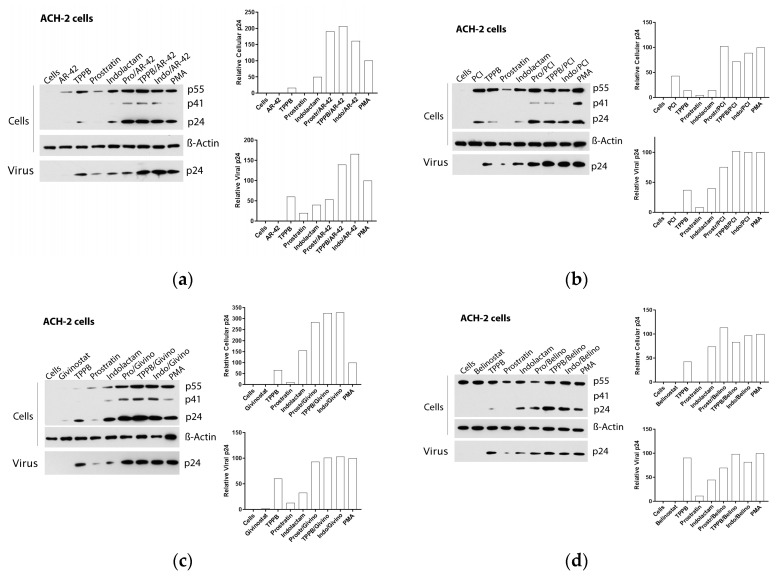
Effect of combinations of LRAs on the reactivation of latent HIV in ACH-2 cells. Immunoblot analysis of cell lysates and viral lysates and related densitometry analysis. β-actin was used as a loading control. The densitometry analysis of the cell-associated Gag p24 is relative to β-actin and expressed as a percentage of the positive control PMA set to 100. The densitometry analysis of the virion-associated Gag p24 is relative to the untreated control cells and expressed as a percentage of the positive control PMA set to 100. Immunoblots are representative of two independent experiments. A representative study of (**a**) AR-42, (**b**) PCI, (**c**) givinostat, and (**d**) belinostat in combination with the PKCas.

**Figure 7:**
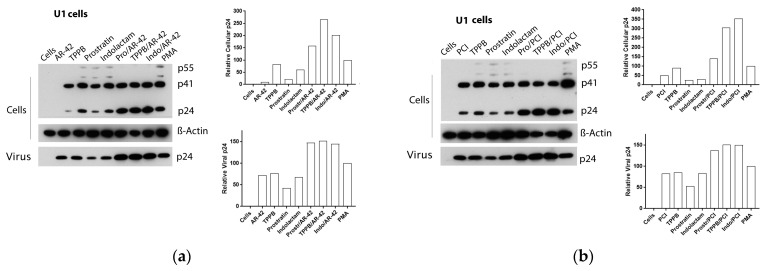

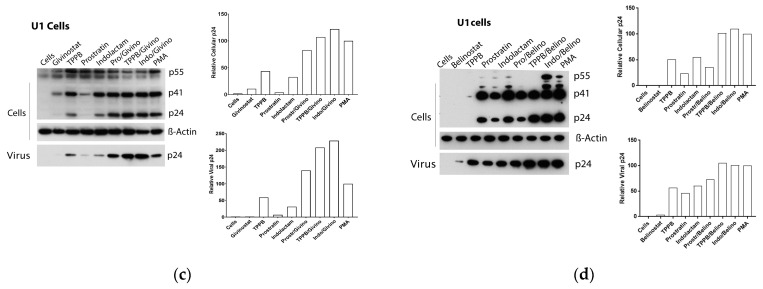
Effect of combinations of LRAs on the reactivation of latent HIV in U1 cells. Immunoblot analysis of cell lysates and viral lysates and related densitometry analysis. β-Actin was used as a loading control. The densitometry analysis of the cell-associated Gag p24 is relative to β-actin and expressed as a percentage of the positive control PMA set to 100. The densitometry analysis of the virion-associated Gag p24 is relative to the untreated control cells and expressed as a percentage of the positive control PMA set to 100. Immunoblots are representative of two independent experiments. A representative study of (**a**) AR-42, (**b**) PCI, (**c**) givinostat and (**d**) belinostat in combination with the PKCas.

Based on these finding, we decided to assess the effect of the LRAs on HIV reactivation by evaluating, in parallel, the HIV RNA expression using real-time qRT-PCR (Figure 4) and viral Gag p24 production using immunoblot (Figure 5, Figure 6 and Figure 7) in both cellular and extracellular viral lysate (culture supernatant). To this end, OM-10.1, ACH-2, and U1 cells were treated with LRAs alone or in combination for 48 h. Previously, it was reported that treatment of OM-10.1 cells with TPA poorly induced HIV reactivation, and higher HIV reactivation was achieved only by exposing these cells to TNFα [36,45,46]. Therefore, we decided to induce OM-10.1 cells only, with TNFα as an additional control. Consistent with other reports [36,45,46], treatment of OM-10.1 cells with PMA induced about 10-fold HIV-1 RNA expression (Figure 4a) with respect to the untreated control, while treatment with TNFα increased about 30-fold the HIV-1 expression with respect to the PMA-treated control (300-fold compared to the untreated control). This resulted in enhanced expression of HIV Gag precursor proteins p55 and p41 and a 12–30-fold increase of gag p24 production, followed by a 1.5-fold increase of extracellular viral RNA and p24, with respect to PMA-treated control (Figure 5a and Figure 4b). Moreover, treatment of OM-10.1 cells with the LRA combinations led to a superior HIV reactivation, and a major buildup of HIV Gag precursor protein p55 and p41, as was observed with the TNFα treatment (Figure 5). The combinations, indolactam/AR-42, TPPB/AR-42, indolactam/PCI, and TPPB/PCI were the most effective, inducing a 40–60-fold increase of the HIV-1 RNA expression compared to the PMA control, and about a 2-fold increase with respect to the TNFα control (400–600-fold compared to the untreated control). This led to a superior expression of the Gag precursors and about 20-fold higher expression of the relative cellular associated p24 compared to the treatment with PMA, and 2-fold higher expression compared to the treatment with TNFα, as shown by the densitometry analysis. However, we only found a 1.2–1.5-fold increase of the extracellular RNA and p24 with respect to the control PMA (Figure 4a,b and Figure 5a,b). Exposing the cells to the combinations prostratin/AR-42 and prostratin/PCI resulted in a lower HIV RNA reactivation (about a 20-fold increase of the HIV-1 RNA expression), which led to Gag precursor and p24 production similar to the previous combinations. Furthermore, in this case, the amount of viral RNA and p24 released in the supernatant were only slightly higher than the control treated with PMA. The combination of givinostat with the three PKCas (Figure 4a,b and Figure 5c) induced a 20-fold increase in HIV RNA reactivation, which resulted in enhanced p55 and p41 expression and a 10-fold increase of p24 production. However, the amounts of viral RNA and p24 released in the supernatant were similar to what we observed for the other combinations. OM-10.1 cells exposed to the combinations of belinostat with the PKCas released similar amounts of viral RNA and p24 in the supernatant even though they induced HIV RNA expression activation and p24 production comparable to the PMA control (Figure 4a,b and Figure 5d).

Exposure of ACH-2 cells to the combinations of TPPB and indolactam with the four HDACis produced a 7–12-fold increase of the cell-associated HIV RNA (Figure 4c,d and Figure 6a–d), while the combination of prostratin with the HDACis was less effective. Treatment with combinations including AR-42, even though it induced a 5–10-fold increase of HIV RNA expression, resulted in a 1.5–2-fold increase of cell-associated p24 and a release of viral RNA and p24 in the supernatant in similar amounts to the PMA control. Comparing with the control treated with PMA, the combinations of the PKCas with PCI induced similar levels of intracellular and extracellular p24, but we noticed a 1.7-fold increase of the extracellular HIV RNA following treatment with TPPB/PCI and indolactam/PCI. This inconsistency may be due to the low sensitivity of the immunoblot. While the combination of givinostat with the PKCas induced HIV RNA expression like the other LRA combinations, it resulted in a 3-fold increase of p24 production with respect to the control treated with PMA. Similarly, they released the same amounts of viral particles as the control PMA, as evidenced by the viral RNA and p24 detected in the supernatant. Combinations of belinostat with the three PKCas induced HIV activation like that observed for the control treated with PMA.

Following treatment with the LRAs, U1 cells shown a lower HIV RNA activation than the other cell lines used in this study, associated with an enhanced expression of the Gag precursor protein p41 (Figure 4e,f and Figure 7a–d). The combinations of LRAs, including TPPB and indolactam with AR-42 and PCI, induced a 1.6–2.1-fold increase of HIV RNA expression with respect to the control treated with PMA, which led to a 2–3.8-fold increase of the Gag p24. These treatments resulted in a 1.5–2.5-fold increase of the extracellular viral RNA and a 1.5-fold increase of the relative viral p24. The combinations, prostratin/AR-42 and prostratin/PCI were less effective at inducing HIV RNA expression. However, in this case, we found enhanced p41 expression and increased amounts of intracellular and extracellular p24 as well as extracellular HIV RNA, similar to the control induced with PMA (Figure 4e). The combinations of belinostat and givinostat with the PKCas induced lower HIV reactivation. Indolactam/givinostat was the only combination that induced higher HIV reactivation than the positive control. Finally, in these cells, we observed that the combinations of indolactam and TPPB with all the HDACis induced a higher viral release in the supernatant than the PMA control, while the combinations including prostratin did not (Figure 4f).

### 3.4. Combinations of PKCas and HDACis Induce High Levels of gp120 Expression

**Figure 8:**
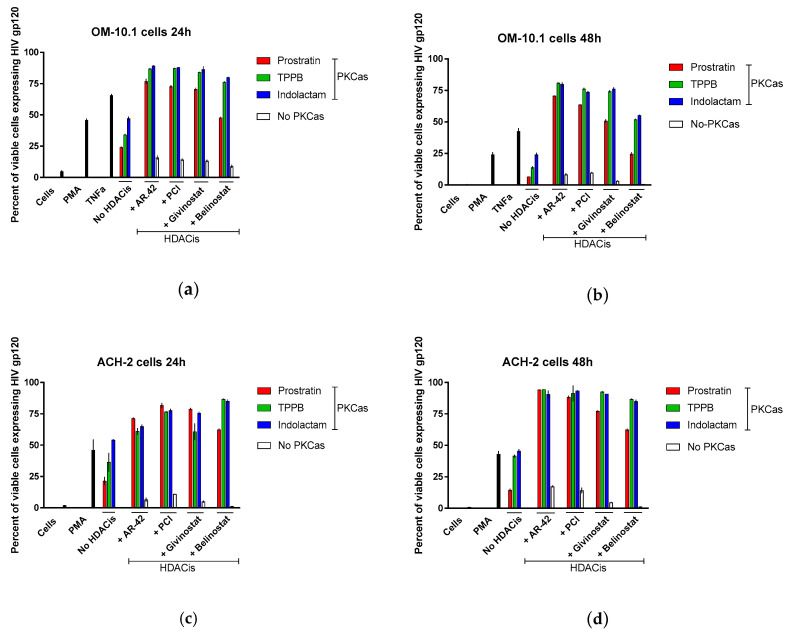
Combinations of LRAs induce gp120 expression in latently infected cells. OM-10.1 cells (**a**,**b**) and ACH-2 cells (**c**,**d**) were treated with LRAs alone or in combination and analyzed for gp120 expression following 24 h and 48 h treatment. OM-10.1 positive control cells were treated with PMA or TNFα. ACH-2 positive control cells were treated with PMA. Cells were incubated with anti-HIV-1 gp120 mAb VRC03, and the gp120 expression was evaluated using flow cytometry by gating on live cells. The percentage of positive cells was reported. Red columns represent cells treated with prostratin in combination with or without HDACis; green columns are cells treated with TPPB in combination with or without HDACis; blue columns are cells treated with indolactam in combination with or without HDACis; and white columns are cells treated with a single HDACi. Data are shown as mean ± S.D. of three independent experiments.

Latently infected cells harboring integrated replication-competent proviruses fail to produce detectable viral protein expression, making them invisible to the immune system. Following stimulation with LRAs, these cells start again expressing viral proteins and produce infectious viral particles [4,5]. Here, we evaluated the expression of envelope glycoprotein gp120 in OM-10.1 and ACH-2 cells following treatment with LRA combinations. Gp120 expression was analyzed using flow cytometry with VRC03 anti-HIV-1 gp120 mAb and gating on live cells (Figure 8). At 24 h, PMA stimulated the expression of gp120 in 50% of the OM-10.1 cells, while TNFα treatment was more efficient, inducing gp120 expression in 70% of cells (Figure 8a). The treatment with the PKCas alone induced gp120 expression in 24–47% of the cells, while treatment with the HDACis was less efficient (8–16% of the cells expressed gp120). Combinations of indolactam and TPPB with AR-42, PCI, and givinostat induced gp120 expression in about 90% of the cells, while the combination of prostratin with the same HDACis induced up to 76%. The combination of belinostat with the PKCas was less efficient (50–80% of cells expressed gp120). Following 48 h treatment, the number of viable cells was less in all the samples, including samples treated with PMA and TNFα, but while in these controls the number of cells expressing gp120 decreased to 24% and 42% respectively, in the combinations including TPPB and indolactam with AR-42, PCI, and givinostat the number of cells expressing gp120 was still 73–80% (Figure 8b). In ACH-2 cells, 24 h of treatment with PMA induced gp120 expression in 46% of the cells only, while all the combinations of LRAs induced gp120 in 62–85% of the cells (Figure 8c). Interestingly, after 48 h treatment, while PMA still induced only HIV expression in 43% of the cells, some of the combinations, including AR-42, PCI, and givinostat, induced gp120 expression in up to 94% of the cells (Figure 8d). Representative flow cytometry of OM-10.1 and ACH-2 cells following 24 h and 48 h treatment with LRAs are shown (Figure S2).

### 3.5. Indolactam/AR-42 Induces CD4 Downregulation

**Figure 9:**
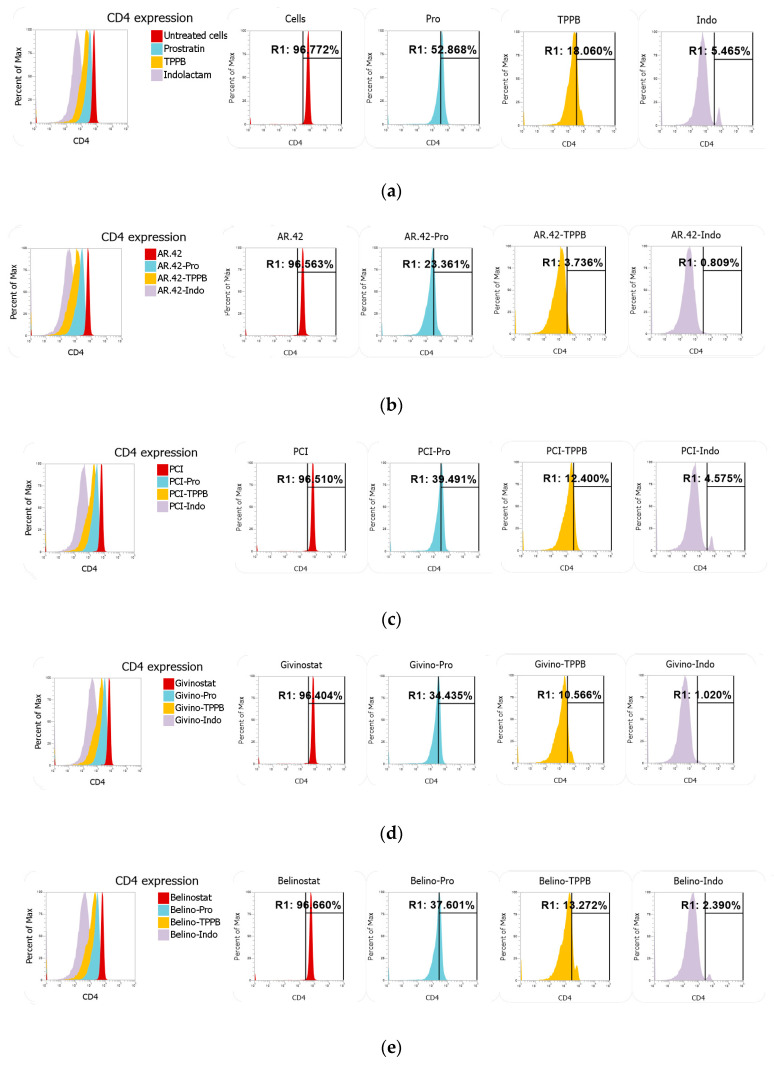
Effect of combinations of LRAs on CD4 expression measured using flow cytometry. Total CD4+ T-cells were stained with anti-CD4 and anti-CD3 following treatment with LRAs for 24 h. (**a**) Representative overlay and single peaks of CD4 expression of untreated cells and cells treated with prostratin, TPPB, and indolactam. (**b**) Overlay and single peaks of CD4 expression of cells treated with AR-42 alone and in combination with PKCas. (**c**) Overlay and single peaks of CD4 expression of cells treated with PCI alone and in combination with PKCas. (**d**) Overlay and single peaks of CD4 expression of cells treated with givinostat alone and in combination with PKCas. (**e**) Overlay and single peaks of CD4 expression of cells treated with belinostat alone and in combination with PKCas.

Many reports have shown that prostratin downregulates the surface expression of CD4, the HIV-1 cellular receptor, through the activation of the protein kinase C (PKC) pathway [24,25,47,48]. Here, we wanted to verify whether TPPB and indolactam, which have not been investigated as extensively as prostratin, show a better profile than prostratin when tested as single compounds or in combination with the HDACis. Total CD4+ T-cells were stained with anti-CD4 and anti-CD3 following treatment with LRAs for 24 h. While the HDACis did not affect CD4 expression, TPPB and indolactam treatment greatly downregulated CD4 expression compared to the untreated control and the cells exposed to prostratin (from about 97% in untreated control and about 53% in cells treated with prostratin to 18% and 5.5% in cells exposed to TPPB and indolactam, respectively) (Figure 9a). Nevertheless, the combination of the PKCas with AR-42 resulted in a more effective downregulation of CD4 expression. For example, following treatment with prostratin/AR-42, 23% of the cells expressed CD4, 3.7% of the cells expressed CD4 following treatment with TPPB/AR-42, and only 0.8% of the cells expressed CD4 when exposed to the combination indolactam/AR-42 (Figure 9b). The other HDACis, in combination with the PKCas, did not enhance the downregulation of CD4 as well as AR-42 (Figure 9c–e). Moreover, we also noticed that while the PKCas and the HDACis alone had no effect on CD3 expression, the combination of these LRAs affected CD3 expression as well (Figure S3). The combinations of AR-42 and belinostat with the PKCas induced surface downregulation of both markers CD4 and CD3, affecting 17.9%, 14.8%, and 21.2% of cells treated with prostratin/AR-42, TPPB/AR-42, and indolactam/AR-42, respectively, and 14.2%, 17.9%, and 25.2% of the cells treated with prostratin/belinostat, TPPB/belinostat, and indolactam/belinostat, respectively. The combinations of PCI and givinostat showed a lower percentage of cells with downregulation of both markers. In fact, the percentage of affected cells were in the range of 6.4–11.5% when exposed to the combinations of PCI with the PKCas and 6.9–10% when exposed to the combinations of givinostat with the PKCas.

### 3.6. Evaluation of HIV Activation in a Primary CD4+ T-Cell Model

**Figure 10:**
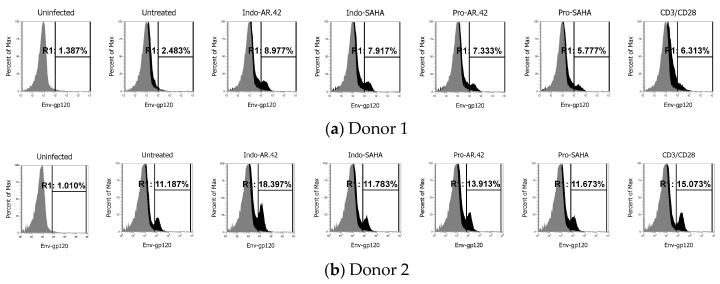
Effect of combinations of LRAs on gp120 expression in HIV latently infected cells. Total CD4+ T-cells were treated with CCL19 and infected with HIV_NL4-3_. Seven days post-infection, the cells were either treated with combinations of LRAs or left untreated, while positive control cells were induced with Dynabeads Human T-Activator CD3/CD28. Cells were incubated with anti-HIV-1 gp120 mAb NIH45–46 G54W, and the gp120 expression was evaluated using flow cytometryfollowing 48 h treatment. (**a**,**b**) Single peak of Env expression of uninfected cells and overlays of Env expression of untreated cells with untreated infected cells and cells treated with the combinations indolactam/AR-42, indolactam/SAHA, prostratin/AR-42, prostratin/SAHA and T-Activator CD3/CD28 from two different donors.

It is known that HIV latently infected cell lines have many limitations and do not reflect HIV latency in primary cells [49]. Many compounds that revert activity in latently infected cell lines do not necessarily revert latency in patient’s cells or a primary cell-based model of HIV latency. In this study, we reported many combinations of LRAs which have shown superior HIV reactivation activity (Table S1). We wanted to validate one of the most effective combinations, indolactam/AR-42, which showed consistent HIV reactivation in all the cell lines tested, combined with an effective CD4 downregulation. We used an in vitro model of HIV latency in primary CD4+ T-cells [18,38,39] as we lack primary CD4 + T-cells from HIV infected patients undergoing ART. We compared the effect of indolactam/AR-42 with combinations of AR-42 and indolactam with two widely investigated compounds, prostratin and vorinostat (SAHA), as follows: indolactam/SAHA, prostratin/AR-42, and prostratin/SAHA. As a control, we exposed the latently infected cells to the human T-cells activator CD3/CD28. We used primary cells from two different donors. Though we detected different percentages of cells expressing gp120 in the untreated control of the two donors, exposing the cells to CD3/CD28 induced an increase of gp120 expression of about 4% with respect to the respective untreated controls (donor 1: from 2.5% to 6.5%; donor 2: from 11.2% to 15.1%) (Figure 10a–b). Moreover, among the combinations we tested, indolactam/AR-42 was the most effective and consistently induced viral reactivation. We observed a 6.3% increase of gp120 expression in donor 1 and a 7.5% increase of gp120 expression in donor 2 when compared with the respective untreated controls. Furthermore, in donor 1, we observed that all the combinations indolactam/SAHA, prostratin/AR-42, and prostratin/SAHA effectively induced viral reactivation with gp120 expression ranging from 5.8% following treatment with prostratin/SAHA to 7.9% with the combination indolactam/SAHA (Figure 10a). By contrast, the same combinations failed or minimally induced HIV reactivation in donor 2 (Figure 10b). These data suggest that the combination of indolactam/AR-42 may effectively induce viral reactivation in primary CD4+ T-cells from HIV infected individuals.

### 3.7. Effect of LRAs Combinations on the Cellular Activation

We finally investigated the effect of the combination of indolactam/AR-42 and the other combinations of LRAs on cell activation and secretion of some inflammatory factors, such as CD69, which is an early activation marker, and HLA-DR, which is a late activation marker. We also investigated CD25, which is the nterleukin-2 receptor alpha chain (IL2RA) and IL-2, and other cytokines and chemokines. Total resting CD4+ T-cells or h-PBMCs were exposed to the combinations of LRAs as well as single LRAs. PKCas are known to induce CD69 upregulation by activating the NF-kB pathway [50]; here as well, we found that 98% of total CD4+ T-cells exposed to PKCas alone or in combination with the HDACis expressed the early activation marker CD69 (data not shown), whereas the CD25 activation marker was expressed in 3.5–8% of the cells exposed to the single PKCas, but it was expressed in only 1–2.6% of the cells exposed to combinations of LRAs (Figure S5a). Moreover, the late activation marker HLA-DR was only expressed in 3–8% of the cells (Figure S5b). The secretion of IL-2 in the supernatant of resting h-PBMCs treated with combinations of LRAs for 48 h was quantified using commercial ELISA tests. We found that the cells treated with LRAs produced IL-2 amounts similar to the quantities produced by the untreated cells (Figure S5c). We also evaluated the intracellular expression of IL-4, IL-12, TNF-α, INF-γ, and CXCL-10 using flow cytometry, and the percentage of cells expressing these factors was 1–5% (data not shown). Following treatment with combinations of LRAs, with the exception of CD69 upregulation, all the other inflammatory markers evaluated in this study were not significantly activated, suggesting that the combinations of LRAs used in this study, including the most effective combination of indolactam/AR-42, do not induce global cellular activation.

## 4. Discussion

The establishment of latency is a complex process involving numerous factors. A combination of agents that target multiple pathways at the same time should be considered as the most successful way to obtain a maximal reactivation of the latent virus. PKC activators and HDAC inhibitors are two important classes of LRAs which have been demonstrated to reverse HIV latency in in vivo clinical trials [12]. In this report, we evaluated the effects of three PKC activators in combination with four HDAC inhibitors. Prostratin is a non-tumorigenic phorbol ester small molecule, largely studied as LRA [23,25,26,48] while (-)-indolactam V [18,19] and TPPB are two benzolactam-derived small molecules, which were not characterized as extensively as prostratin. Moreover, AR-42 and PCI-24781 are also two HDACis poorly characterized as LRAs. Our results show that combinations of LRAs successfully induced HIV-1 reactivation in all the cell lines we used and in a primary CD4+ T-cell model. The use of different cell types was necessary to demonstrate that a good combination of LRAs is capable of reactivating viral protein expression in different cell models, as latently infected patients carry viral DNA integrated into different chromosomal locations [41,42]. The low cytotoxicity levels observed with the PKC agonists as single compounds, and in combination with the HDAC inhibitors, encourage the use of higher doses to achieve higher levels of HIV-1 reactivation. We observed that the proliferation rate and the percentage of cells expressing activation markers were higher in cells treated with PKCas alone than in cells exposed to combinations of LRAs (Figure 2 and Figure S4). On the other hand, the HDACis alone had a bigger impact on cell viability, but no effect on cellular activation than the combinations of LRAs. The data suggest that the combinations of HDACis with the PKCas were in some way attenuating the unfavorable toxic effects of the single HDACis and the cellular activation/proliferation induced by the PKCas. However, the mechanism of this attenuation is yet to be elucidated. We presented in this study that the most effective HIV latency reactivators were AR-42 and PCI in combination with indolactam and TPPB. These combinations showed synergistic effects exceeding the additive effects of the same compounds when administered separately as measured by GFP expression in the two J-Lat cell lines and p24 release in the U1 and ACH-2 cell lines (Figure S1 and Figure 3). These findings were further confirmed using quantitative real-time RT-PCR and immunoblot. The data showed that the combination of AR-42 and PCI with indolactam and TPPB induced higher amounts of intracellular viral RNA compared to the positive control PMA (up to a 12-fold increase in ACH-2 cells and up to a 60-fold increase in OM-10.1 cells, which corresponded to a 1.2–1.9-fold increase of HIV expression to the cells exposed to TNFα, which was used for maximal HIV induction) (Figure 4). This activation resulted in a major enhancement of Gag protein expression; however, at the same time, the amount of viral particles released in the supernatant was similar to what we observed for the controls induced with PMA or TNFα. Moreover, combinations of AR-42, PCI, and givinostat with TPPB and indolactam efficiently reactivate viral expression and exposure of the surface envelope glycoprotein gp120 following 24 h treatment (up to 90% in OM-10.1 cells and up to 85% in ACH-2 cells). This is an important combination of events since HIV latently infected cells are invisible to the immune system as they fail to produce detectable viral protein expression. LRA combinations induce a massive viral protein expression, including the envelope gp120 making these cells visible and susceptible to the natural or engineered immune response. However, at the same time, the release of a moderate amount of viral particle could easily be contained to avoid a de novo infection.

PMA and prostratin and other PKC agonists are known to downregulate CD4 [24,25] and CD3 [51,52] expression. Moreover, the treatment with HDACis could also have a negative influence by affecting the cytotoxic T-lymphocyte (CTL) functions and the elimination of infected CD4+ T-cells [53]. Prostratin, TPPB, and indolactam downregulated CD4 expression, but the same concentrations used in this study did not affect CD3 expression. However, the effect of these compounds on CD4 expression was enhanced by the combination with the HDACis. Furthermore, these combinations also caused the downregulation of CD3 in some of those cells. While the HDACis alone did not affect CD4 and CD3 expression, we noticed that especially AR-42 clearly potentiated the effect of the PKCas on the expression of both markers. Similar results were reported by Walker-Sperling et al. [52] who found that HDACi romidepsin potentiated the effect of PKCa bryostatin-1 by transiently downregulating the CD3 expression of unstimulated CD8+ T-cells, thereby causing a delay in eliminating the reactivated CD4+ T-cells. Since combinations of these LRAs could result in a delayed or decreased T-cell response, studies to assess their effect on CD8+ T-cells are ongoing. On one side, the transient downregulation of CD3 and TCR complex could result in the delay of the immune response, which in the shock and kill setting could be a negative factor. At the same time, CD4 downregulation could be considered as an advantage, considering that one of the major concerns with the shock and kill strategy is that uninfected CD4+ T-cells will become infected by HIV as it is purged from latent reservoirs. Previous reports have shown that over 93% of proviruses in resting CD4+ T-cells in HIV+ individuals undergoing cART are defective, and the majority of virus produced is noninfectious [54,55]. Additionally, HDACis such as belinostat and givinostat have been reported to induce the decrease of HIV release from macrophages by the degradation of intracellular HIV through the canonical autophagy pathway [56]. Our findings support the previous reports. In fact, the combinations of PKCas with the HDACis induced a massive intracellular HIV reactivation with respect to the PMA or TNFα controls; however, the amounts of viral particles released by these cells were similar to the controls. Since the concentrations of PKCas used in this study did not affect CD3 expression, and the combination with the HDACis seemed to potentiate their effect, one pathway to avoid the CD3 downregulation and delayed immune response will be to adjust the concentrations of both classes of compounds to achieve the highest HIV activation, without interfering with CD3 expression, and obtain a reduction of adverse effects in general, such as toxicity and inflammation. One encouraging example of the outcome of the adjustment of the concentrations of two LRAs, prostratin and AR-42, in combination is reported (Figure S4). We found that maintaining the same concentration of prostratin while lowering AR-42 concentration significantly reduced the CD3 downregulation.

LRAs that revert activity in latently infected cell lines do not necessarily revert latency in patient’s cells or primary cell-based models of HIV latency [49]. Here, we identified indolactam/AR-42 as one of the most effective latency-reversing combinations based on the consistency it has shown reactivating HIV from latency through all cell lines and assays, combined with an effective CD4 downregulation. We validated the efficacy of this combination in an in vitro model of HIV latency in primary CD4+ T-cells [18,38,39], and we compared it with the activity of well-known compounds, prostratin and SAHA (Figure 10). Indolactam/AR-42 was the most efficient and consistent combination in reversing HIV latency, as shown by the 6.5% and 7.5% increase of gp120 expression. In contrast, the other combinations showed lower efficacy and consistency; in fact, they had minimal or absent activity when tested in donor 2.

## 5. Conclusions

We reported our concerted effort in identifying the best combinations of LRAs using widely reported PKCas and HDACis. We identified indolactam/AR-42 in our study as one of the most effective combinations, as evidenced by the results showing the maximal HIV reactivation and gp120 expression in both cell lines and HIV infected primary CD4+ T-cells, low toxicity and low cell activation in uninfected cells, and low viral release and higher CD4 downregulation, which could be an advantage by reducing de novo infection during the shock and kill strategy. Indolactam/AR-42, with the unique combination of major latency-reversing effect and downregulation of CD4, showed its potential as a possible candidate for a more in-depth study with latently infected memory CD4+ cells isolated from HIV infected patients who were under treatment for a long period. Further experiments will also be necessary to fully understand the effects of this LRA combination.

## Figures and Tables

**Table 1:** Evaluation by dose response of HIV activation (EC_50_) using ELISA (OM-10.1, U1, and ACH-2 cells) and GFP expression (J-Lat 10.6 and J-Lat A2 cells) and cytotoxicity (CC_50_) of LRAs after 48 h treatment.

Compound	Target	OM-10.1		U1		ACH-2		J-Lat 10.6		J-Lat A2	
		EC_50_^1^	CC_50_^1^	EC_50_^1^	CC_50_^1^	EC_50_^1^	CC_50_^1^	EC_50_^1^	CC_50_^1^	EC_50_^1^	CC_50_^1^
Prostratin	PKC	0.41 ± 0.03	>8	0.3 ± 0.03	>8	0.31 ± 0.07	>8	0.68 ± 0.01	>8	0.87 ± 0.03	>8
TPPB	PKC	0.13 ± 0.01	>8	0.21 ± 0.01	>8	0.07 ± 0.01	>8	0.51 ± 0.08	>8	0.49 ± 0.03	>8
(-)-Indolactam V	PKC	0.09 ± 0.02	>8	0.12 ± 0.02	>8	0.18 ± 0.03	>8	0.2 ± 0.04	>8	0.32 ± 0.05	>8
Belinostat	HDAC	0.88 ± 0.4	2.7 ± 0.2	0.77 ± 0.01	>8	0.44 ± 0.03	1.1 ± 0.2	0.94 ± 0.03	1.4 ± 0.06	0.94 ± 0.05	1.4 ± 0.02
PCI-24781	HDAC	0.35 ± 0.04	0.4 ± 0.02	0.46 ± 0.03	~8	0.37 ± 0.04	0.31 ± 0.02	0.45 ± 0.02	0.4 ± 0.02	0.38 ± 0.05	0.54 ± 0.03
Givinostat	HDAC	0.45 ± 0.13	0.42 ± 0.01	0.63 ± 0.02	~4	0.32 ± 0.04	0.63 ± 0.03	0.88 ± 0.04	0.93 ± 0.04	0.7 ± 0.05	1.2 ± 0.02
AR-42	HDAC	0.53 ± 0.08	0.4 ± 0.01	0.26 ± 0.1	~8	0.24 ± 0.04	0.41 ± 0.01	0.55 ± 0.02	0.45 ± 0.02	0.44 ± 0.05	0.53 ± 0.05

^1^ The reported EC_50_ and CC_50_ values represent the means ± standard deviation (SD), n = 3. Values are expressed as µM.

## Supplementary Materials

The following are available online at https://www.mdpi.com/1999-4915/12/6/609/s1, Figure S1: Effect of LRAs in combination on the reactivation of latent HIV measured using flow cytometry, Figure S2: Effect of combinations of LRAs on HIV gp120 expression following 24 h and 48 h treatment. Figure S3: Effect of combinations of LRAs on CD4 and CD3 expression measured using flow cytometry. Figure S4: Evaluation of combinations of different concentration of prostratin and AR-42 using flow cytometry. Figure S5: Effect of LRA combinations on cellular activation. Table S1. Comparison of the effect of the LRA combinations on different experimental conditions.
