# Supplementary material for: Identification of Combinations of Protein Kinase C Activators and Histone Deacetylase Inhibitors that Potently Reactivate Latent HIV
Source: Viruses. 2020 Jun 3;12(6):609. doi: 10.3390/v12060609 (PMC7354613; doi:10.3390/v12060609)
Supplement: Supplementary file 1 [file viruses-12-00609-s001.pdf]

Supplementary Materials

Effect of LRAs in combination on the reactivation of latent HIV measured by flow cytometry. We evaluated the activity of combinations of LRAs in J-Lat A2 and J-Lat 10.6 cells. Cells were treated with LRAs alone or in combination for 48 h. As a control, cells were untreated or induced with PMA. Exposure of J-Lat 10.6 cells (**Figure S1a**) to HDACis alone induced about 2-10% GFP expression with respect to the PMA control, while the PKCas alone induced higher levels of GFP expression (37-86%). The combination of Prostratin with the four HDACis produced a synergistic effect. The synergy/antagonism effect of drug combinations was analyzed with the Bliss independence model *[1]*. When used alone, Prostratin activated 37% of GFP expression, but the combination of Prostratin/AR-42 induced 70.4% of GFP expression (AR-42 induced 10.2% when tested alone), 68% in the combination Prostratin/PCI (PCI alone induced 6.5%) and 57.7% in the combination Prostratin/Givinostat (Givinostat induced 2.9% when tested alone). The combination Prostratin/Belinostat was the least efficient inducing about 48.5% of GFP expression. A similar synergistic effect was also observed with the combination of TPPB with all the four HDACi and, also in this case, we observed that the combination with Belinostat was the least effective to activate GFP expression. Moreover, we observed a synergistic effect of the combinations of Indolactam with AR-42, PCI, and Givinostat, respectively, but there was an antagonistic effect when Indolactam was combined with Belinostat. Representative flow cytometry plots of GFP expression in J-Lat 10.6 cells for the combination of PCI with the three PKCas are shown in **Figure S1c**. Furthermore, in the J-Lat A2 cells (**Figure S1b**) we observed that the single HDACis induced higher amounts of GFP expression (10-35%) than in J-Lat 10.6 cells, while the PKCas induced similar levels of GFP expression (37-79%). All the combinations induced GFP expression with a synergistic effect. The combinations of the three PKCas compounds with AR-42, PCI, and Givinostat induced levels of GFP expression higher than what was observed in the positive control treated with PMA. Additionally, the combination of the PKCas with Belinostat did not induce higher levels of GFP expression than PMA, even though there was a clear synergistic effect. Representative flow cytometry plots of GFP expression in J-Lat A2 cells for the combination of AR-42 with the three PKCas are shown in **Figure S1d.**

Figure S1. Effect of LRAs in combination on the reactivation of latent HIV measured by flow cytometry. (a) (c) J-Lat 10.6 cells, and (b) (d) J-Lat A2 cells. The number of GFP-positive cells was expressed as a percentage of the positive control PMA set to 100 (black columns). Red columns represent cells treated with Prostratin in combination with or without HDACis; green columns are cells treated with TPPB in combination with or without HCDAis; blue columns are cells treated with Indolactam in combination with or without HDACis and white columns are cells treated with a single HDACi. Data are shown as mean ± S.D. of three independent experiments. (c) Representative flow cytometry of GFP expression in J-Lat 10.6 cells treated with PCI alone or in combination with Prostratin, TPPB, and Indolactam. Also shown the GFP expression in untreated cells, cells treated with PMA and cells treated with the single three PKCas as controls. (d) Representative flow cytometry of GFP expression in J-Lat A2 cells treated with AR-42 alone and in combination with Prostratin, TPPB, and Indolactam. Also shown the GFP expression in untreated cells, cells treated with PMA and cells treated with the single three PKCa as controls.

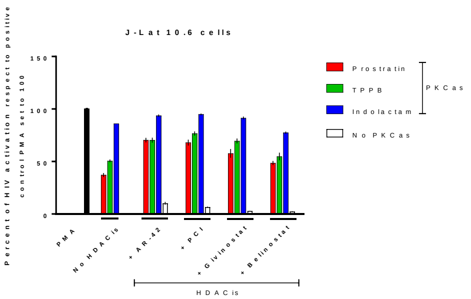

(a)

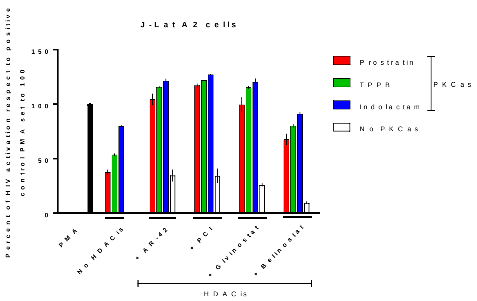

(b)

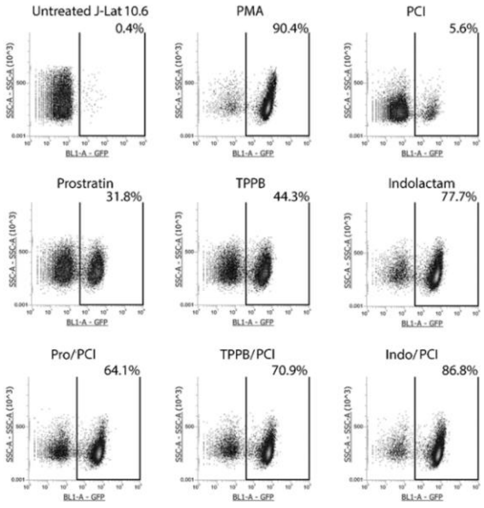

(**c**)

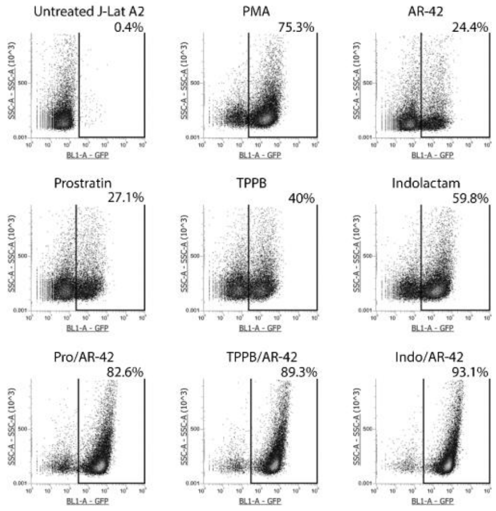

(**d**)

Figure S2. **Effect of combinations of LRAs on HIV envelope glycoprotein gp120 expression following 24 h and 48 h treatment.** Representative flow cytometry of OM-10.1 cells (c) and ACH-2 cells (f) following 24 h and 48 h treatment with LRAs.

Envelope gp120 expression in OM-10.1 cells (24 h treatment)

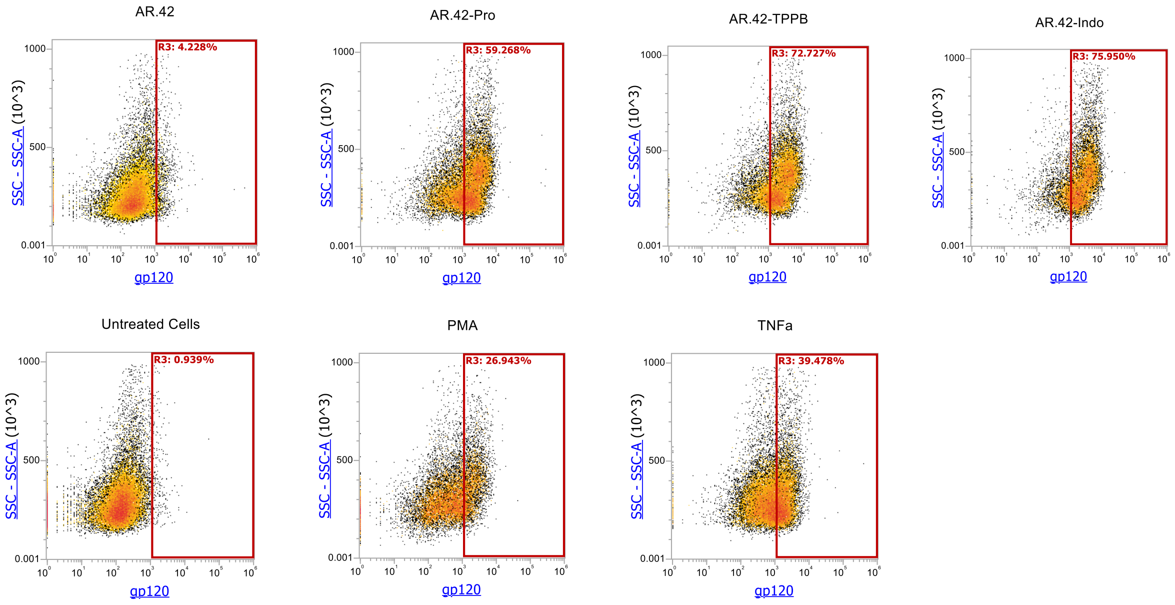

Envelope gp120 expression in OM-10.1 cells (48 h treatment)

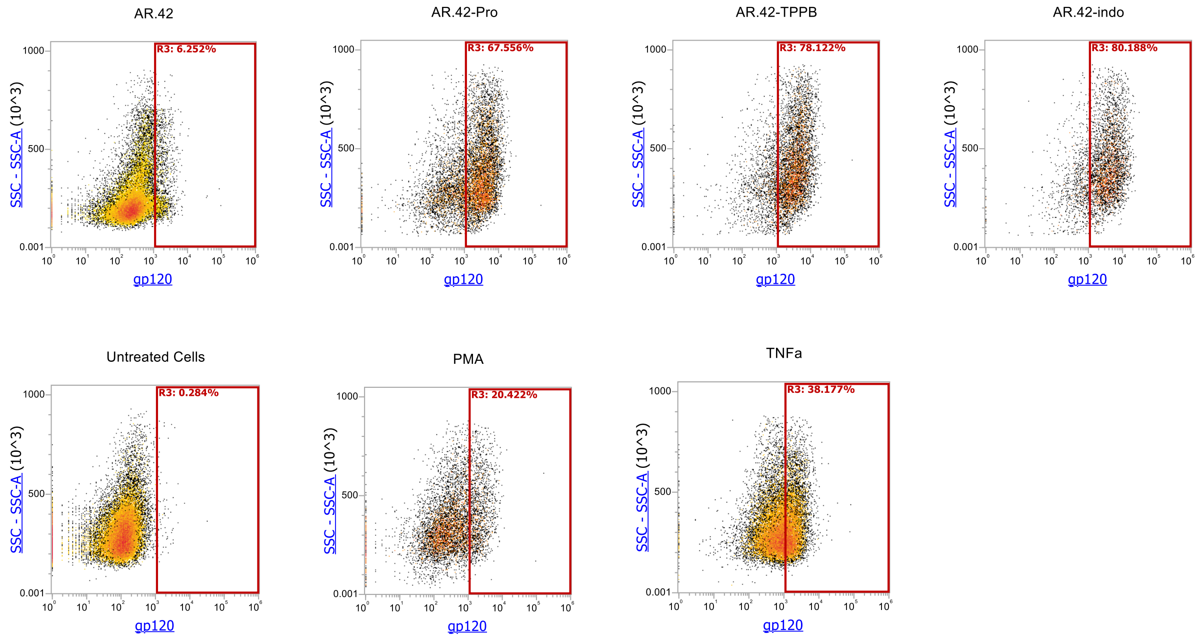

Envelope gp120 expression in ACH-2 cells (24 h treatment)

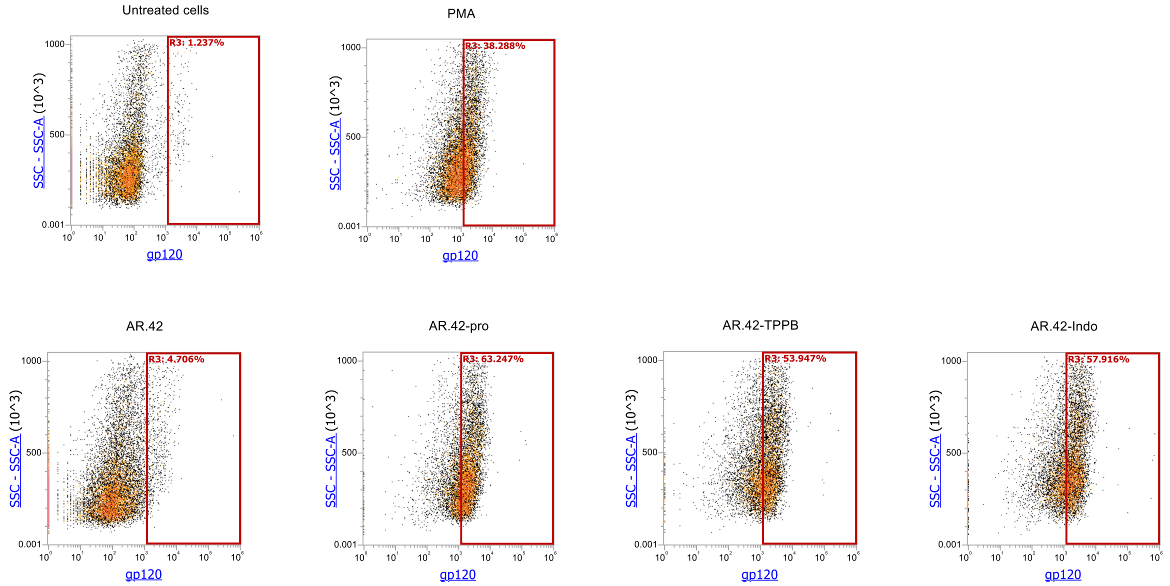

Envelope gp120 expression in ACH-2 cells (48 h treatment)

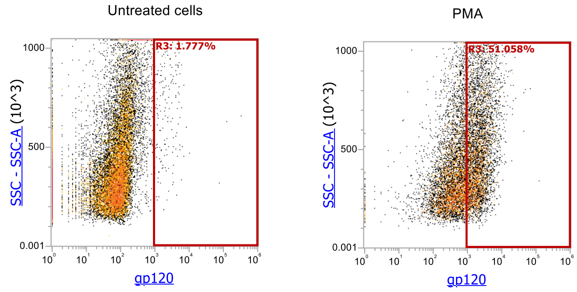

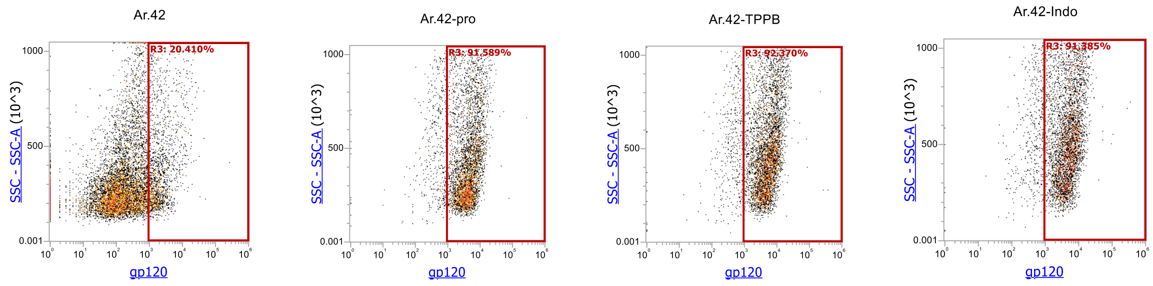

Figure S3. Effect of combinations of LRAs on CD4 and CD3 expression measured by flow cytometry. Total CD4+ T cells were stained with anti-CD4 and anti-CD3 following treatment with LRAs for 24 h. Representative Flow cytometry of CD4 expression of untreated cells, cells treated with LRAs alone or in combination.

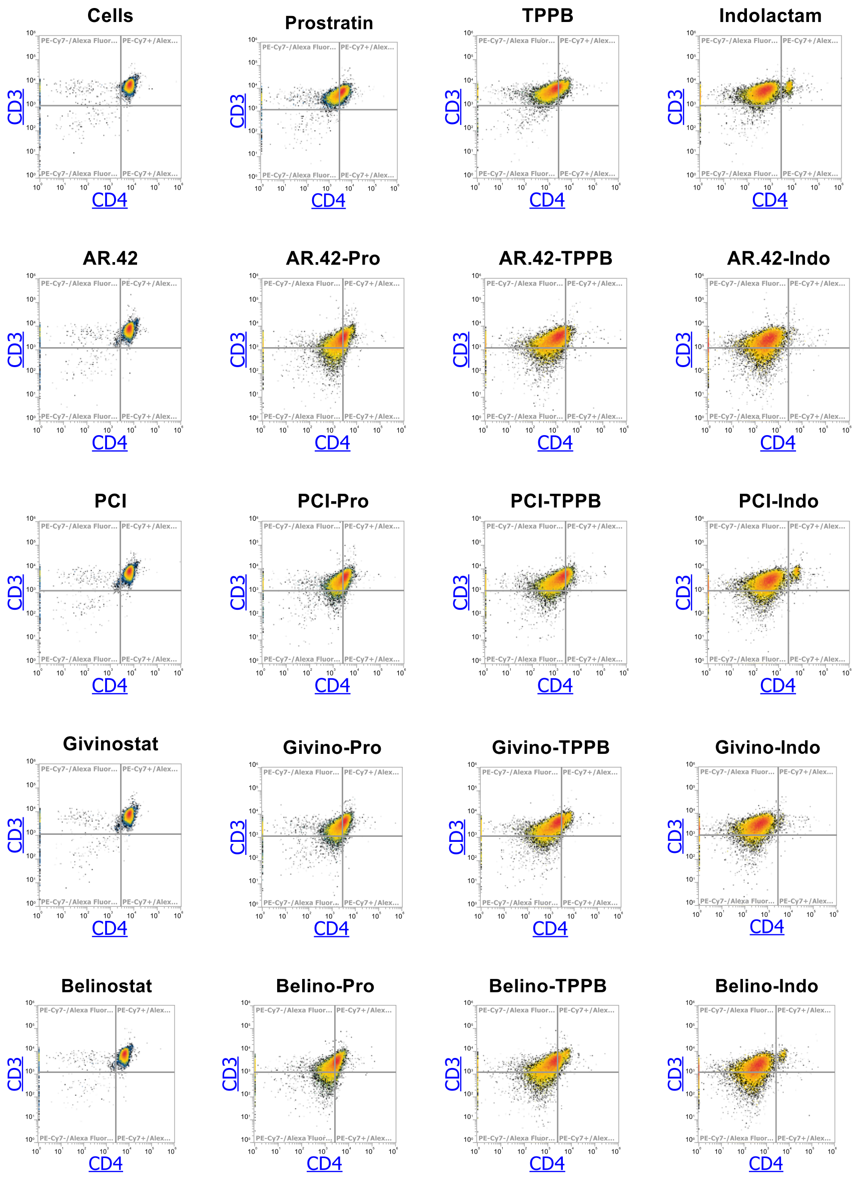

Figure S4. Evaluation of combinations of different concentration of Prostratin and AR-42 by flow cytometry. We performed a second study using different dosages of Prostratin and AR-42 in combination. Once more, we found that AR-42 alone did not affect CD4 and/or CD3 expression while, as expected, Prostratin downregulated CD4 expression but did not have any impact on CD3 expression. When 0.3 μM of AR-42 was combined with different concentrations of Prostratin, we noticed that the cell population expressing CD4 decreased compared to Prostratin alone, suggesting that AR-42 enhanced the effect of prostratin Moreover, AR-42 (0.3 μM)/Prostratin (0.5 μM) downregulated the expression of both CD3 and CD4 in 45% of these cells. When we kept the concentration of Prostratin at 0.5 μM and reduced AR-42 concentration (Prostratin 0.5 μM/AR-42 0.2 μM and Prostratin 0.5 μM/AR-42 0.1 μM) we observed that the number of cells affected by downregulation of both CD4 and CD3 was dose-dependent (26.5% and 5% respectively), however, the downregulation of CD4 remained unchanged. This data suggests that to avoid CD3 downregulation, it will be necessary to maintain Prostratin at higher concentrations and lower the concentration of AR-42.

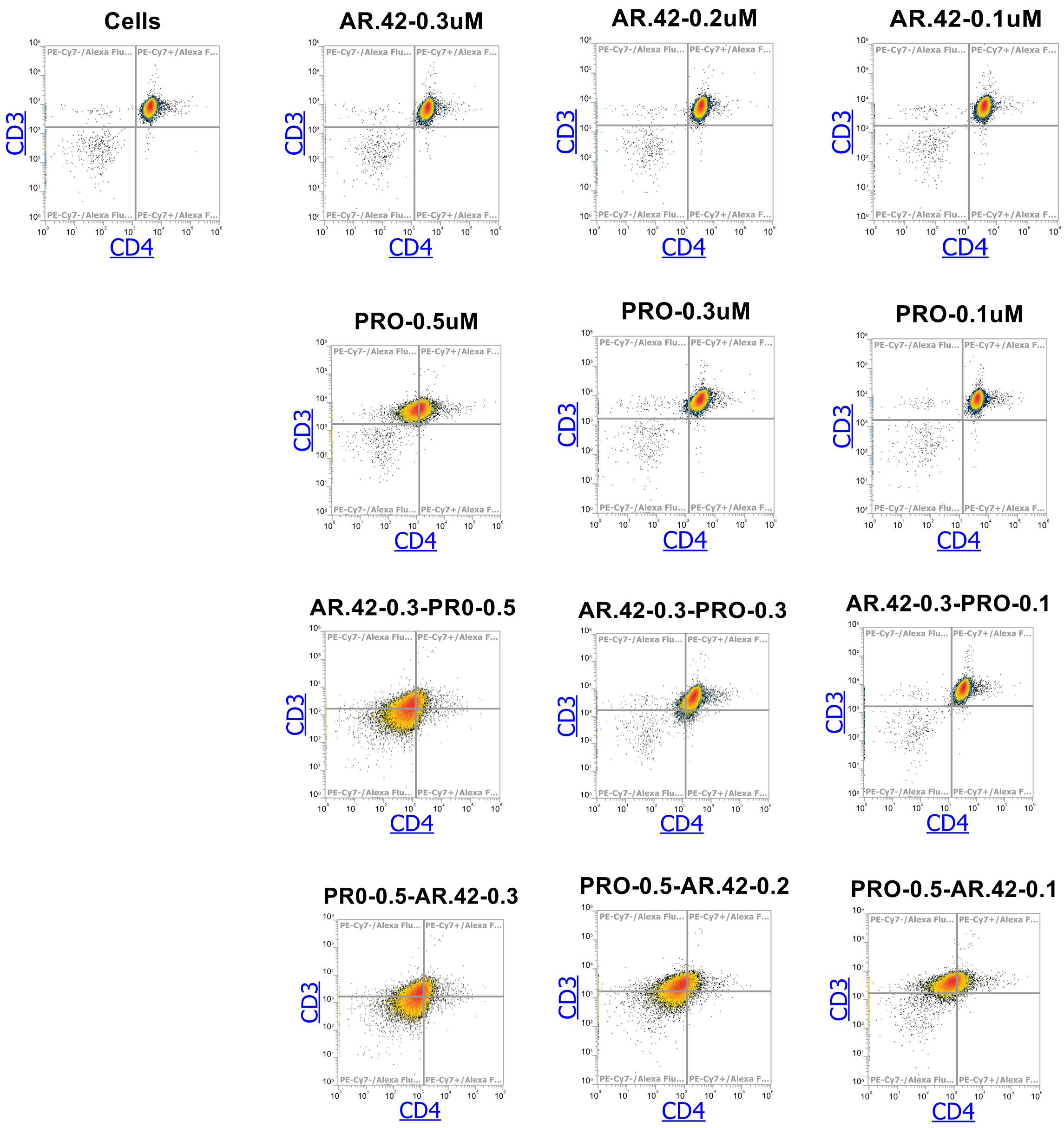

Evaluation of cellular activation

Methods: **ELISA for detecting IL-2**. Total resting CD4+ T cells were plated at 10^6/ml in 96-well plate and treated with LRAs alone or in combination. As a control, we used untreated cells and cells induced with Dynabeads Human T-Activator CD3/CD28 (Thermofisher). Following 48 h treatment, the supernatants were collected and mixed with an equal volume of 5 % Triton X-100 and tested for IL-2 antigen by ELISA using the Human Interleukin-2 (IL-2) uncoated ELISA kit (Invitrogen) by following the manufacturer's instructions. The GraphPad Prism software was used to interpolate the OD values obtained for the samples from the standard linear curve. Total CD4+ T cells from two different patients were analyzed in triplicate.

Detection of CD69, CD4, CD25 and HLA-DR, DP, DQ expression by flow cytometry. Total resting CD4+ T cells were plated and treated with LRAs alone or in combination. As a control, cells were untreated or induced with the Dynabeads. Following 48 h treatment, the Dynabeads were removed from the control group and the plate was spun to pellet the cells and remove the supernatant. Cells were washed with PBS and incubated with the mAbs in 50 µl staining buffer for 20 min. Following two washes with PBS cells were resuspended with 200 µl PBS and evaluated by flow cytometry. The anti-CD69 mAb (FN50), Super Bright 600 was purchased from eBioscience™, the anti-CD4 mAb (S3.5) and anti-CD3 mAb (UCHT1) were from Invitrogen and the anti-CD25 and anti-HLA-DR, DP, DQ from Miltenyi Biotec. Total CD4+ T cells from three different donors were analyzed in triplicate.

Cytokines detection. H-PBMC cells were plated at $2x10^6$/ml and treated with LRAs alone or in combination. As a control, cells were untreated or induced with the Dynabeads as above. Brefeldin A was added 4 h prior to flow cytometry analysis. Cells were washed with PBS and incubated with Fixable Viability Dye eFluor™ 450 (eBioscience™) for 30 min. Then the cells were fixed and permeabilized using the intracellular fixation & permeabilization buffer set (eBioscience™) following the manufacturer's instructions. Cells were stained with labeled mAbs and incubated for 1 h. Finally, the cells were washed 2X and resuspended with 200 µl PBS. The cytokines expression was evaluated by flow cytometry. The Abs used in this assay are CXCL10 (IP-10) mAb (4NY8UN), TNF-α mAb (MAb11), IL-4 mAb (8D4-8), IFN gamma mAb (4S.B3) (eBioscience™) and IL-12 p35 mAb (Invitrogen).

Figure S5. Effect of LRAs combinations on the cellular activation. Total resting CD4+ T cells were treated with combinations of LRAs as well as single LRAs for 24 h and evaluated for (a) HLA-DR activation and (b) CD25 expression by flow cytometry. (c) The secretion of IL-2 in the supernatant of resting h-PBMC treated with combinations of LRAs for 48 h was quantified by commercial ELISA tests. Data are shown as mean ± S.D. of three independent experiments

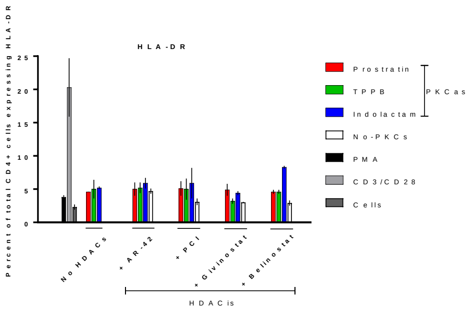

(a)

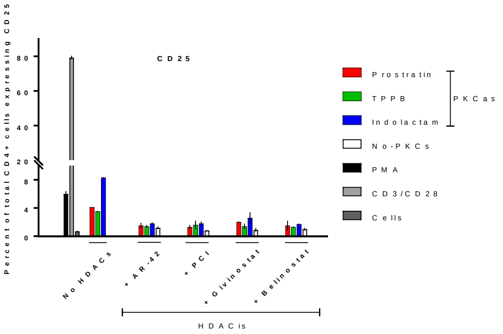

(b)

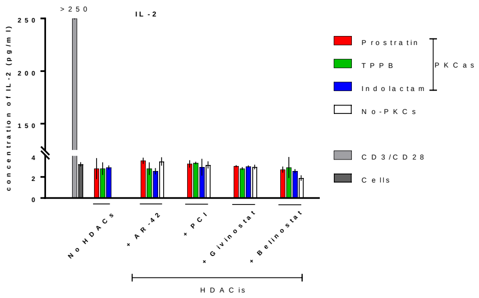

(c)

Table S1. Comparison of the effect of the LRAs combinations on different experimental conditions. Expression of envelope protein gp120 (flow cytometry), glycoprotein p24 release (immunoblot) and CD4 expression (flow cytometry)

	% Gp120 expression **OM-10.1** 24 h / 48 h	% Gp120 expression **ACH-2** 24 h / 48 h	% P24 release (WB) 48 h **OM-10.1** / **ACH-2** / **U1**	% CD4 expression **Total CD4+ Tcells** 24 h
Pro/AR-42	76.8 / 70.6	71.3 / 94.1	132.4 / 53 / 147.5	23.4
TPPB/AR-42	86.9 / 80.9	61 / 94.4	112.8 / 139.7 / 152	3.7
Indo/AR-42	89.3 / 79.8	64.9 / 90.7	128.1 / 165.5 / 145	0.81
Pro/PCI	72.7 /63.6	81.8 / 88.4	121.8 / 75.3 / 138.1	39.5
TPPB/PCI	87.3 / 76.2	76.6 / 91.3	141.8 / 101.5 / 150.6	12.4
Indo/PCI	88 / 73.7	77.6 / 93.3	132.6 / 100 / 150	4.6
Pro/Givino	70.6 / 50.8	78.8 / 77.2	111.5 / 93.3 / 140.2	34.4
TPPB/Givino	84.1 / 74.3	60.7 / 92.5	101.3 / 101.1 / 208.4	10.6
Indo/Givino	86.4 / 76.2	75.6 / 90.9	100.6 / 102.8 / 228.9	1
Pro/Belino	47.8 / 24.6	62.4 / 62.4	60.2 / 69.9 / 72.9	37.6
TPPB/Belino	76.3 / 51.9	86.7 / 86.7	78 / 98.5 / 105.5	13.3
Indo/Belino	80 / 55.3	85 / 85	159.1 / 81.8 / 101	2.4

References

40. Foucquier, J.; Guedj, M. Analysis of drug combinations: Current methodological landscape. *Pharmacol. Res. Perspect.* **2015**, *3*, e00149, doi:10.1002/prp2.149.
